# Emerging Bioanalytical Devices and Platforms for Rapid Detection of Pathogens in Environmental Samples

**DOI:** 10.3390/mi13071083

**Published:** 2022-07-08

**Authors:** Lightson Ngashangva, Bahaa A. Hemdan, Mohamed Azab El-Liethy, Vinay Bachu, Shelley D. Minteer, Pranab Goswami

**Affiliations:** 1 Transdisciplinary Biology, Rajiv Gandhi Centre for Biotechnology (RGCB), Thiruvanthapuram, Kerala 695014, India; ng.lightson@rgcb.res.in; 2 Department of Biosciences and Bioengineering, Indian Institute of Technology Guwahati, Guwahati, Assam 781039, India; be.hemdan@iitg.ac.in (B.A.H.); vinay.2015@iitg.ac.in (V.B.); 3 Water Pollution Research Department, Environmental and Climate Change Research Institute, National Research Centre, 33 El Buhouth Street, Cairo P.O. Box 12622, Egypt; ma.el-liethy@nrc.sci.eg; 4 Department of Chemistry, University of Utah, 315 South 1400 East, RM 2020, Salt Lake City, UT 84112, USA

**Keywords:** pathogens, biosensors, bioanalytical devices, smart materials, responsive materials

## Abstract

The development of robust bioanalytical devices and biosensors for infectious pathogens is progressing well with the advent of new materials, concepts, and technology. The progress is also stepping towards developing high throughput screening technologies that can quickly identify, differentiate, and determine the concentration of harmful pathogens, facilitating the decision-making process for their elimination and therapeutic interventions in large-scale operations. Recently, much effort has been focused on upgrading these analytical devices to an intelligent technological platform by integrating them with modern communication systems, such as the internet of things (IoT) and machine learning (ML), to expand their application horizon. This review outlines the recent development and applications of bioanalytical devices and biosensors to detect pathogenic microbes in environmental samples. First, the nature of the recent outbreaks of pathogenic microbes such as foodborne, waterborne, and airborne pathogens and microbial toxins are discussed to understand the severity of the problems. Next, the discussion focuses on the detection systems chronologically, starting with the conventional methods, advanced techniques, and emerging technologies, such as biosensors and other portable devices and detection platforms for pathogens. Finally, the progress on multiplex assays, wearable devices, and integration of smartphone technologies to facilitate pathogen detection systems for wider applications are highlighted.

## 1. Introduction

The ever-increasing cases of various diseases in humans and animals caused by microbial pathogens such as bacteria and viruses are a serious concern in medicine. One of the difficulties in dealing with most infectious pathogens is their unpredictable nature, as they may contaminate from various sources and infect at any time. Therefore, it is imperative to detect the pathogens as early as possible to contain their dissemination. Typically, pathogens are detected using conventional techniques and methodologies, such as microscopy, polymerase chain reaction (PCR)-based protocols, culture and colony counting, and immunological methods [1]. Although reliable and sensitive, these methods encounter many drawbacks; they are complex, time-consuming, expensive, require skilled operators, and, most importantly, challenging to deploy in resource-limited environments and locations that require onsite monitoring. With time, portable, low-cost diagnostic devices such as biosensors have received increasing popularity among the public, as they facilitate rapid pathogen detection in samples and self-diagnosis of various diseases and monitoring health-related aberrations (such as for diabetics). Recently, the COVID-19 pandemic has greatly increased the interest in such diagnostic devices among individuals and across various government and NGO agencies for rapid monitoring and management of the disease in broad community structures [2]. For developing such devices, multidisciplinary approaches offer practical solutions, as these approaches improve the detection efficiency, dimensional requirement, data exchange among the stakeholders, cost economy, and public perception of the products. In this context, molecular biology, nanotechnology, advanced and smart materials, communication technology, and microscale technology, including microfluidics, micro-electromechanical system (MEMS), lithography, 3D printing, and design, have played significant roles in development and commercialization of biosensor products [3]. Since the inception of micro-total analysis systems a few decades ago, many sensitive and selective miniaturized diagnostic devices for various infectious pathogens have been developed [4]. Articles focusing on different specific aspects of the subject, such as rapid pathogen detection methods [5], electrochemical detection for viruses [6], biosensors for foodborne pathogens using the microfluidic platform [7], point-of-care (POC) for waterborne pathogens [8], livestock and poultry [9], methodologies for pathogen and toxins [10], and point-of-care microfluidic devices for pathogen detection [11] are available. However, this comprehensive review focuses on the latest developments on the subject, particularly on the technological front over the last five years. This review also highlights the technical gaps that need to be bridged for developing a robust biosensor for infectious microbial pathogens.

## 2. Pathogens and Microbial Toxins

### 2.1. Foodborne Pathogens

Annually, 600 million cases of foodborne illnesses are reported, out of which 420,000 people die each year [12]. Foodborne pathogens can grow in an environment containing various nutrients and continuously spread in the surrounding environment [13]. They are commonly found in contaminated food and dairy products and directly impact public health. The population of such pathogens can drastically increase in some disastrous events, such as floods and tornadoes [14]. At least 31 foodborne pathogens are bacteria, viruses, or parasites. The common foodborne bacteria are *Clostridium botulinum*, *Clostridium perfringens*, *Bacillus cereus, Campylobacter* spp., *Brucella* spp., various strains of *E. coli* (e.g., O157, non-O157, Enterotoxigenic, Diarrheagenic), *Listeria monocytogenes*, *Mycobacterium bovis*, *Yersinia enterocolitica, Salmonella enterica* serotype Typhi, *Salmonella* spp., *Shigella*, *Staphylococcus*, *Streptococcus* group A, *Vibrio cholerae*, *Vibrio vulnificus,* and *Vibrio parahaemolyticus* [15]. Consumed foods characterize foodborne pathogens; for instance, *Salmonella* is usually present in poultry, eggs, and meat products. *Campylobacter* spp. are found with undercooked poultry; *Shigella* spp. and enteropathogenic *E. coli* are often found with milk and meat foodstuffs. *Clostridium botulinum* is present in home-canned foodstuffs. Furthermore, *Yersinia* spp., *Staphylococcus aureus*, *Listeria* spp., *Clostridium perfringens, Vibrio cholerae, V. vulnificus*, *V. parahaemolyticus*, and *Bacillus* spp. are present in raw milk and uncooked meats, in addition to vegetables [16]. Some foodborne bacteria such as *Clostridium* and *Bacillus* spp. are heat resistant. The majority of these bacteria are mesophilic (ranging from 20 to 45 °C). The others are psychrotrophic or psychrophilic (grow at < 10 °C), such as *Listeria monocytogenes* and *Yersinia enterocolitica* [17]. The major foodborne viruses are astrovirus, hepatitis A, norovirus, and rotavirus. These viruses cannot survive for a long time outside the host. Finally, most parasites causing foodborne diseases are *Cryptosporidium* spp., *Cyclosporacayetanensis*, *Giardia intestinalis*, *Toxoplasma gondii,* and *Trichinella* spp. 

### 2.2. Waterborne Pathogens

Contaminated water is a transmission source for many disease-causing pathogens. Potable water and wastewater may contain a vast array of opportunistic microbial pathogens [18]. Water-related diseases might result from water overload or shortage, sanitary sewage, and drinking water treatment plants. Moreover, pathogenic microorganisms may also be transmitted into groundwater, surface water, and recreational water (such as swimming pools, springs, hot tubes, spas, and fountains) [19]. Drinking water contamination depends on the population of microbial pathogens [20], the nature of the delivered water, the age of the pipelines [21], and climatic changes that overload the treatment plant tasks [22].

There are different pathogenic microbes transmitted to human hosts through contaminated water. Some widely known bacterial pathogens are *Burkholderia pseudomallei, Campylobacter* spp., *E. coli* (diarrhoeagenic and enterohaemorrhagic), *Francisella*, *Legionella pneumophilia*, *Mycobacterium avium*, *Salmonella typhi*, *Salmonella enterica*, *Salmonella bongori*, *Shigella dysenteriae*, and *Vibrio cholerae* O1 and O139. Among the waterborne viruses, adenovirus, astrovirus, norovirus, sapovirus, hepatitis A and E viruses, enterovirus, parevirus, and rotavirus are reported as pathogens. Some known waterborne protozoa are *Acantha moabacubertsoni*, *Cryptosporidium hominis*, *Crytospordium parvum*, *Cyclospora cayetanensis*, *Entamoaba histolytica*, *Giardia intestinlis* and *Naegleria fowleri*, in addition to Helminths [23]. Some of the known entrance routes are through skin, digestion (fecal-oral route), inhalation, and direct contact with the mucous membranes of the nose, ear, mouth, eye, and genital organs [24]. Inhalation of contaminated aerosol drops may cause respiratory infections, e.g., pneumonia and sepsis, by water-related pathogens such as *Legionella* spp. Water-related pathogens such as *P. aeruginosa, Aeromonas* spp., *Acinetobacter* spp., and *Naegleria* spp. may cause local infection and sepsis through the skin and mucous membranes of the ear, nose, and eyes [25].

Due to waterborne pathogens in drinking water, approximately 1.4 million deaths of children are reported every year globally [26]. Protozoa infections transmitted through water are the second causative agent for deaths among children younger than five years old. Worldwide, protozoa are the leading cause of 1.7 billion diarrheal illnesses, leading to 842,000 deaths each year, as per the studies conducted during the period 2011–2016 [27]. Globally, ~200 million people are suffering from Schistosomiasis, though it is not endemic. In 2011, around 1060 waterborne parasite cases of Guinea worm disease caused by *Dracunculus Medinensis,* were reported, especially in areas with no safe drinking water. Malaria, another protozoal disease, can be spread by mosquitos breeding in polluted surface water and infects about 300 to 500 million individuals, with more than one million deaths per year, as per the studies conducted during 2011–2016 [27].

### 2.3. Airborne Pathogens

Airborne microbial pathogens are transmitted from the infected hosts to the susceptible hosts via various paths, such as aerosolization, sneezing, breathing, coughing, or even during conversation. They spread in the air via aerosols (small particles ≤ 5 µm), droplets (large particles > 5 µm), dust particles, or spores [28]. The most common airborne viruses are measles, norovirus, influenza A (H1N1), avian influenza (H5N1 and H7N9), rubella, SARS, rhinovirus, and *varicella-zoster*. The most known airborne bacteria are *Mycobacterium tuberculosis*, *Acinetobacter baumannii*, *Clostridium difficile*, *Bordetella, Bacillus antiphrasis*, *Streptococcus pneumonia,* and *Legionella* spp. The most recorded airborne fungi are *Aspergillus* spp., *Penicillium, Cladosporium, Stachybotrys, Fusarium, Cryptostroma,* and *Blastomyces dermatitidis* [29]. In 1918, the H1N1 influenza virus (also named Spanish influenza) infected ~500 million people worldwide, of which 50–100 million died. In 2009, the H1N1 strain of pig influenza spread quickly around the globe among people. The WHO recognized it as a pandemic disease and considered it a non-zoonotic influenza virus, meaning not transmitted from pigs to humans. This virus can spread via airborne droplets from human to human through contact [30]. Another airborne virus is avian influenza, which includes many subtypes (H5N1, H5N2, H3N2, and H7N9). It mainly infects poultry and livestock and might be transmitted to people [31]. Rubella virus causes German measles, and it spreads through droplets by sneezing or coughing [32]. In 2003, a member of the coronavirus family causing severe acute respiratory syndrome (called SARS) infected more than 8000 people, causing 774 deaths [33]. Later in December 2019, the novel SARS-CoV-2 strain (COVID-19) that originated from Wuhan city rapidly spread [34], infecting > 505,267,277 and killing > 6,224,938 people globally (https://www.worldometers.info/coronavirus/, accessed on 19 April 2022).

Numerous bacterial pathogens, such as *Streptococcus pneumoniae, Bacillus anthracis,* and *Mycobacterium tuberculosis* commonly cause many airborne diseases [35]. *M. tuberculosis* is a Gram-positive bacterium that causes meningitis, pneumonia, and tuberculosis (TB) [36]. TB is a highly infectious disease with a low infectious dose of about ten colony forming units (CFU) of *M. tuberculosis.* It is reported that *M. tuberculosis* infects about 14 million people and causes approximately 2 million deaths per year, as per the census carried out for 1990–2021 [37]. *B. anthracis* is a Gram-positive bacterium that causes anthrax in humans, but it is not communicable. The mortality rate of *B. anthracis* is still high, reaching up to 45% [38]. *S. pneumoniae* is a Gram-positive and aerobic bacterium and can cause several airborne sicknesses, such as sinus infections, meningitis, pneumonia, and septicemia. Likewise, ten common serotypes of *S. pneumonia* are responsible for causing more than 60% of the world’s bacterial diseases [39].

Some fungi are reported as airborne pathogens, such as Cladosporium, Penicillium, Mucor, Aspergillus, Cryptostroma, Alternaria, Fusarium, Stachybotrys, and Absidia [40]. Aspergillus causes aspergillosis in people with debilitated resistant frameworks or lung illnesses. Among fungi, Blastomyces dermatitidis is an exceptionally pathogenic dimorphic organism in both soggy soil and decayed matter [41].

### 2.4. Microbial Toxins

Many microbial pathogens produce toxins that can promote infection and cause diseases by destroying the host tissues and suppressing the host immune system. The toxin size may range from small- to macro-molecules, such as peptides and proteins [42]. There are many bacterial toxins related to water or foodborne sicknesses. For instance, *Vibrio cholerae* O1 and O139 produce cholera toxin, Enterotoxigenic *E. coli* produces thermolabile toxin and thermostable toxin. Both *Shigella dysenteriae* and *E. coli* O157:H7 produce Shiga toxin, *Clostridium perfringens* secretes CPE enterotoxin, *Clostridium difficile* produces both A and B toxins, and *Bacillus cereus* produces cytotoxin K (CytK) [43]. The most potent bacterial toxins known at present are Botulinum neurotoxins. *Clostridium botulinum* (*C. botulinum)* can produce eight exotoxins, namely A, B, C_1_, C_2_, D, E, F, and G. Such exotoxins can cause muscle paralysis, breathing problems, double vision, and muscle weakness in the infected hosts by blocking neurotransmitter production [44].

Fungal toxins (mycotoxins) are the most abundant toxins in the environment [45]. Mycotoxins are secondary organic metabolites and are synthesized by several fungi, such as *Fusarium, Penicillium,* and *Aspergillus* [46]. Humans are exposed to mycotoxin particles by ingestion, inhalation, and skin contact, and they enter the blood and lymphatic streams to cause serious diseases such as liver damage, tumor growth, inflammation, and immunosuppression [47]. The United Nations in 2016 noted that almost 25% of the global food crops contain mycotoxins [48]. The most prominent mycotoxins are mycotoxins (AFs), ochratoxin, trichothecenes, zearalenone, and fumonisins, including FB1 and FB2 [49]. Aflatoxins are harmful secondary metabolites, produced by fungi such as *Aspergillus* spp. Aflatoxins. These toxins are usually found in agricultural crops as the fungi infect crops such as wheat, corn, peanuts, etc. Approximately 4.5 billion persons are exposed to infection from consuming foodstuffs contaminated by aflatoxins [50].

Aflatoxin M1 (AFM1) is commonly found in milk. The AFM1 is a hydroxylated metabolite of AFB1 and has carcinogenic and immunosuppressive effects [51]. Many algae from marine environments, such as cyanobacteria, commonly known as blue-green algae, produce various biotoxins, including microcystins or cyanoginosins, that have detrimental effects on humans and other animals. Microcystins (MCs) are distributed in tissue by organic anion transport proteins (OATPs). Because of excessive expression of OATPs in the liver, MCs could be considered hepatotoxins. However, the distribution of MC is not limited to the liver but also to other organs. It is observed that MC has a high binding affinity with protein phosphatases; such interactions would hamper many biological regulatory pathways, such as cell replication, DNA repair or stress response, and cytoskeletal structure [52]. Microcystins can cause hepatotoxicity and long-term tumorigenicity in humans. As the number of algal blooms around the world has increased, the frequency and intensity of harmful toxin production have also surged, which is a matter of concern [53]. The algal biotoxins may cause nausea, headache, gastrointestinal disturbance, respiratory malfunctions, and neurological disorders [54].

## 3. Pathogen Detection Systems

**Figure 1:**
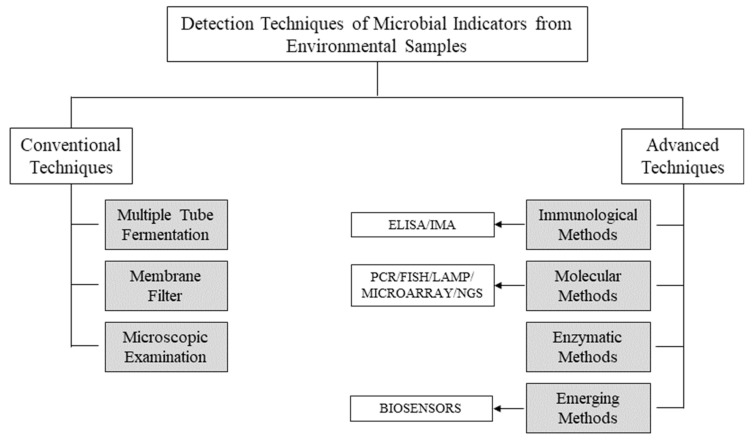
Schematic diagram for conventional and advanced laboratory-based techniques used for monitoring microbial pathogens in environmental samples. ELISA: enzyme-linked immunosorbent assay; IMA: immunomagnetic assay; PCR: polymerase chain reaction; FISH: fluorescent in-situ hybridization; LAMP: loop-mediated isothermal amplification; NGS: next generation sequencing.

Microbial detection approaches are typically divided into phenotypic methods, such as culture-based and molecular techniques, such as DNA-based assays. The culture-based approach is commonly recommended for testing and detecting infectious pathogens due to its affordability and simplicity. Surveillance programs are commonly conducted using culture-based methods that demand an incubation period of 1–4 days based on the target organisms’ types. This long detection time required to produce definitive results significantly impedes their implementation [55]. The traditional culture-independent methods for pathogen detection, such as qPCR, offer results in less time (Figure 1) [56].

### 3.1. Conventional Techniques

Traditional approaches depend on metabolic activity reactions or a growing response in an adequate substratum after a sufficient incubation period. Some of the standard investigation approaches are multiple tube fermentation (MTF), membrane filter (MF), and microscopic techniques (Figure 1).

#### 3.1.1. Multiple Tube Fermentation

In this method, also known as the most probable number (MPN) method, an appropriate volume of the environmental sample is transferred into tubes with the required broth media and then placed in the incubators. These procedures involve an estimation of bacterial cells’ densities using mathematical statistics and, therefore, do not provide absolute values. By mixing chromophore or fluorophore, or a mixture of both in the broth medium as marker agents, the conventional methods can be customized for a particular group, genera, or species. These approaches save time as these target species are distinguished by specific enzymes that change the color of the media during substrate hydrolysis [57].

#### 3.1.2. Membrane Filter

Membrane filter (MF) techniques can be used to identify the microorganisms in aqueous environmental samples. Through suction, the aqueous sample is filtered, and in this process, microorganisms are trapped in the membrane filter. The membrane is carefully positioned on a suitable agar plate, and the passage of nutrients facilitates the microorganisms’ growth to form colonies on the membrane surface. These colonies may be transferred to confirmation media, and, following the incubation, they are counted using optical microscopy techniques. These techniques are relatively low-priced, convenient to use, and require no specialized machinery. These approaches, however, are non-specific, so determining the source of contamination is a challenge [25].

However, these culture-dependent techniques are not suitable for detecting extremely slow-growing bacteria and bacteria with viable-but-not-culturable (VBNC) types [58].

#### 3.1.3. Microscopic Examination

Microscopy is another conventional technique that can be used independently or in conjunction with other conventional techniques. It is a rapid method for the diagnosis of infectious diseases. These methods, such as Gram, Ziehl–Neelsen, and fluorophore stain, remain the most easily available and viable techniques in various clinical setups. A brief description of the advantages and limitations of the conventional techniques are presented in Table 1.

### 3.2. Advanced Techniques

Advanced techniques help to overcome many drawbacks of the traditional methods. These techniques offer a high level of sensitivity and selectivity, affordable prices, and a wide range of indicators for pathogenic microbes [59]. However, they do not differentiate live from dead bacteria and need intensive preparation and highly sophisticated facilities. The benefits and drawbacks of the advanced techniques are summarized in Table 2.

#### 3.2.1. Immunological Methods

Many detection techniques rely upon antigen–antibody complex reactions, such as enzyme-linked immunosorbent assay (ELISA) and immunomagnetic assay (IMA). ELISA is a cellular component-based method that employs chromogen or fluorogen with a specific enzyme [66]. Antibodies are labeled with a colorant that emits the fluoresced light under UV radiation. The development of color intensity in the reaction can be correlated with the densities of microbial species. The time of assay for bacteria ranges from 1 to 2 days. However, some samples may require enrichment steps to ensure reliable identification [67]. After filtering the aqueous environmental sample, the quantification can also be performed by epifluorescence microscopy or solid-phase cytometry. Even though numerous ELISA kits are available commercially, there is still a demand for suitable kits for aquatic ecosystem applications [68].

#### 3.2.2. Nucleic Acid-Based Methods

Nucleic acid-based techniques employ probe molecules such as DNA and RNA that can detect particular molecular genetic fingerprints for specific microbial strains or groups of microbes. Some techniques include polymerase chain reaction (PCR), fluorescence in-situ hybridization (FISH), and isothermal strategies, such as isothermal amplification induced by the loop (LAMP) [69].

##### PCR-Based Methods

This is the most widely used molecular-based approach for identifying pathogens. By targeting unique DNA sequences, PCR facilitates the identification of the pathogenic microbes [70]. A particular DNA sequence is multiplied in a cyclic three-step process: denaturation, annealing, and expansion. The accuracy of identifying the target DNA of microbes in an environmental sample is significantly improved by PCR cycling [71]. The PCR-based technique has been applied to determine waterborne pathogens such as *E. coli*, enterotoxigenic *E. coli,* and *C. perfringens* spores [72]. Even though the benefits of PCR technology are evident, it has several drawbacks [73]. The key downside of PCR-related strategies is the long reaction time and minimal functionality because they depend on thermal cycling and need additional equipment to distinguish amplified sequences. Further, these techniques need technical experts, hampering the deployment of PCR-based systems in resource-limited settings and onsite applications. Additionally, the simple version of the technique cannot differentiate live from dead bacterial cells, and false positives results may be generated under some conditions. An advanced variant of PCR is the real-time PCR (RT-PCR) technique. RT-PCR is considerably more accurate. In this approach, the amount of DNA amplicons is being measured and quantified at each specific time or cycle, so the technique is also called quantitative PCR or qPCR. The other variations of PCR methods, such as reverse transcriptase PCR (using RNA rather than DNA), have been established to minimize the limitation of conventional PCR methods. The reverse transcription–polymerase chain reaction is a commonly used semiquantitative approach in medical science and research, such as in nanotoxicology investigations [74].

##### Fluorescence In-Situ Hybridization (FISH) Method

In the FISH strategy, specific nucleic acid sequences within viable cells are detected using oligonucleotide probes. Epi-fluorescence microscopy is generally used to assess the stained cells after hybridization and post-hybridization washing [75]. Currently, it is regarded as a precise and simple technique for cellular detection. The technique’s sensitivity depends on the oligonucleotide probe and the parameters being applied during the hybridization. It identifies viable cells such as *E. coli* [76]. FISH requires pre-enrichment or pre-concentration steps, which may give false-negative results due to the insertion of potential inhibitors in the sample. A standard plate count is the most widely used approach for estimating the number of living bacteria in environmental samples. However, plate count is generally many orders of magnitude less than the actual number of living bacteria present in the sample; hence, assessing the quantity of viable cells by this method is limited. It is worth noting that most bacteria in environmental samples are in a “viable but non-culturable” (VBNC) state, and may be resuscitated when appropriate conditions are provided. During the last decade, approaches other than FISH have also been employed to track the VBNC bacteria, such as immunological procedures, qPCR, and the commercial kit LIVE/DEAD^®^*Bac*Light™ assay [77].

##### LAMP-Based Method

Loop-mediated isothermal amplification (LAMP) is one of the widely employed amplification techniques. Though it is less versatile than PCR, it has received significant research interest due to its good precision, high amplicon concentrations, and low cost. The method can be performed at a specific temperature, simplifying the detection process and allowing better portability than PCR-based methods [78]. The method can detect *E. coli, Proteus hauseri, Vibrio parahemolyticus,* and *Salmonella* subspecies with high sensitivity and selectivity. Microchips preloaded with agarose solution containing LAMP reagents, when stored at 4 °C, can be used for 30 days, facilitating the long-term storage and transport of LAMP reagents necessary for point-of-care applications [79]. However, even though the technique could be used for the absolute measurements of bacterial targets, including *E. coli* and *S. typhi*, the appropriate detection limit has not been adequately documented [80].

##### DNA Microarray

Considering the significance of analyzing a large number of samples with speed and accuracy, strategies such as DNA microarrays and next-generation sequencing have been applied to detect pathogens. The microarrays platform is a useful genomic tool used in environmental samples to examine gene expression under various cell proliferation conditions, diagnose unique mutations in DNA sequences, and classify microbes [81]. The analyses are conducted in an ordered two-dimensional matrix of immobilized high-density nucleic acids (genomic DNA or oligonucleotides), allowing hundreds of genes to be identified simultaneously in a single reaction through the nucleic acid hybridization process [82]. This technique has great potential to identify pathogen characteristics and their origins and has already been applied to bacterial detection [83]. Another example is the PhyloChip phylogenetic microarray sold by Affymetrix, consisting of 500,000 oligonucleotide probes that detect 8743 species of bacteria and archaea [84].

##### Next-Generation Sequencing (NGS)

NGS is an innovative technology that can perform massively parallel sequencing and determine the order of nucleotides either in the whole genome or in DNA or RNA regions. Sequencing analysis of different regions as one of the primary aspects of microbial assemblages in the small subunit rRNA detects distinct microbial species that can act as markers for pathogens. NGS can also provide high-quality screening of sequences to identify contaminants. Shrestha et al. [85] indicated that *Acinetobacter, Arcobacter*, and *Clostridium* were recently identified as potential pathogenic bacteria using 16S rRNA gene NGS. Genomic DNA was cloned from different samples entering the 16S rRNA gene V4 region, and pathogenic species were described by comparing the sequences with a reference human pathogenic bacteria database [86]. In general, most modern approaches are now under review, and before being generally usable, they require standardization and validation.

#### 3.2.3. Enzymatic Methods

Enzymatic approaches focus on identifying particular enzymes involved in the targeted microbes’ primary metabolic pathway. The detection relies mostly upon the color or fluorescence change in the enzymatic reaction. Chromogenic substances are incorporated to observe the color changes, and fluorescent images can be observed under UV light if a fluorescent dye is tagged in the method [87]. Such an enzymatic approach with the defined-substrate technique is utilized to decrease the prolonged assay period of traditional approaches. As such, to target a single bacterium, the indicator substances are specially engineered and designed. Various enzymatic substrates, such as Colilert^®^, Enterolert^®^, m-ColiBlue^®^, ColiComplete^®^, and Chromocult^®^, could be utilized in enzyme-based methods [88]. Moreover, the enzyme-specific substrates can be used directly in environmental samples and incubated without harvesting under optimal conditions for the enzyme in question. The level of cell numbers of microbial species in environmental samples can be assessed quickly using enzymes such as β-D-galactosidase or β-D-glucuronidase with fluorogenic substrates [89].

## 4. Biosensors for Pathogen Detection

The biosensor is an analytical device in which a bio/molecular recognition element is coupled to a transducer and a signal processor. The device generates readable and quantifiable signals corresponding to the physico-bio-chemical attributes of the target analyte/sample. Biosensors offer many advantages, such as being quick, sensitive, selective, portable, affordable, and simple-to-operate, among others, as compared to conventional complex and sophisticated detection systems [90]. Biosensors have been undergoing rapid advancement recently in terms of function and affordability. The devices can enable real-time monitoring and diagnosis of diseases, even to the extent of prediction, and allow for preventive measures while combating various pathogenic microbes [91]. Functional improvement could be possible with the intervention of various advance and smart materials in the fabrication processes. *Smart materials* may be defined as materials that are responsive to stimuli, such as pH, light, electrical, mechanical, or chemical changes, or pressure, without an external influence [92]. With the advancement of science and technology in general and smart materials development in particular, detection systems have been significantly improved [93]. The application of smart material platforms in detection systems has expanded from single-molecule/target to multiple target analytes/molecules in various samples, including detection of pathogens in environmental samples [94]. Some known smart materials in biosensing applications for infectious pathogens are nanomaterials, molecularly imprinted polymers (MIPs), hydrogels, photonic crystals, ionic liquids (ILs), and responsive polymers.

### 4.1. Nanomaterials Based Systems

Some biosensors employ nanomaterials to enhance performance, improve sensitivities and improve the limit of detection (LOD). Nanomaterials such as metal nanoparticles (NPs), quantum dots (QDs), carbon nanomaterials, and polymer nanoparticles (PNPs) are extensively studied for the detection of pathogens, including foodborne ones [95], bacteria, and viruses [96]. They are usually employed through a non-covalent approach (π-π stacking, hydrogen bonding, trap, van der Waals interaction, electrostatic interaction, and others), which can retain all the properties of nanomaterials and biomolecules. In contrast, due to uncontrolled fixation and interaction, the covalent approach (amide bonding, crosslinking, clicking chemistry, etc.) may affect the properties. Below are some examples of recent development (within the last five years) of nanomaterial-based biosensors for pathogenic microbes.

**Figure 2:**
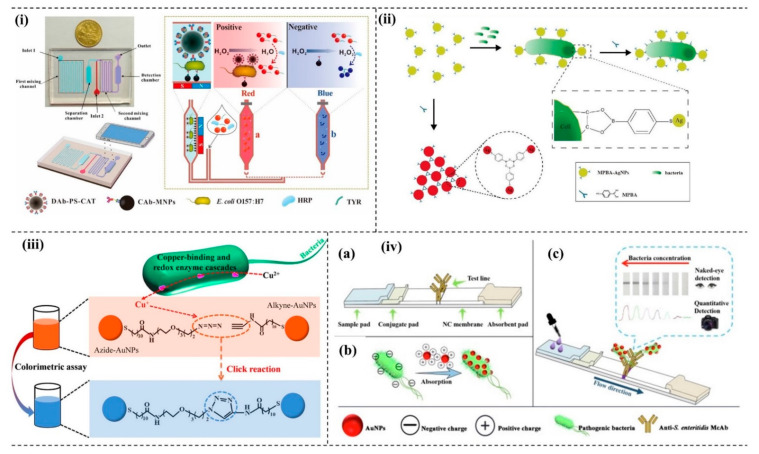
Metallic nanomaterials-based pathogen detection system: (**i**) *E. coli* O157:H7 is detected by tuning the optical property of AuNPs by using tyramine as a crosslinking agent in microfluidic platform. Reprinted with permission from Ref. [97]. Copyright 2019 Elsevier. (**ii**) Colorimetric detection of bacteria explores the aggregation and inhibition of aggregation of MPBA-AgNPs. Reprinted with permission from Ref. [98]. Copyright 2018 Elsevier. (**iii**) Aggregation of AuNPs is instructed by bacteria to undergo click chemistry and triggered in the presence of Cu^+^. Reprinted with permission from Ref. [99]. Copyright 2019 American Chemical Society. (**iv**) Schematic representation of the colorimetric assay of *S. enteritidis* based on positively charged AuNPs using lateral flow technology, (**a**) test strip structure, (**b**) interaction of (+) AuNPs- (−) *S. enteritidis* mechanism, (**c**) colorimetric and quantitative detection of *S. enteritidis* [100].

Gold nanoparticles (AuNPs) exhibit unique physical and chemical properties, which can be tuned to realize the desired function of a biosensor. The optical property may be modulated by tuning the size of nanoparticles (NPs). Further, the optical properties of the monodispersed and aggregated NPs are different and are the basis for color-based detection of various analytes. A microfluidic-based biosensor for *E. coli* 0157:H7 was developed by incorporating AuNPs in the device. The method exploited smartphone imaging technology to capture the color change of AuNPs to determine the concentrations of *E. coli* 0157:H7 [97]. The detection principle of the developed biosensor involved AuNP aggregation. A crosslinking agent such as tyramine (TYR) assists the aggregation of AuNPs that change color from blue to red (Figure 2i). The phenolic hydroxyl group of TYR activates the aggregation of the AuNPs. The average diameter sizes of NPs before and after the addition of tyramine are 13 nm and ~ 670 nm, respectively. The compound 4-mercaptophenylboronic acid-functionalized silver nanoparticle (MPBA-AgNPs) was also used to rapidly detect bacteria using biosensors. The MPBA-AgNPs aggregate in the presence of an excess of MPBA. However, in the presence of bacteria, the MPBA-AgNPs are dispersed and could give different colors, as shown in Figure 2ii [98]. The presence of Cu^+^ between a functionalized azide and alkyne AuNPs triggers the interesting click chemistry that leads to the aggregation of NPs and thus changes the red color to blue, as shown in Figure 2iii. In this study, Cu^2+^ was initially reduced by the pathogenic bacteria to Cu^+^, which triggered the click-chemistry between the modified AuNPs. Through this strategy, the colorimetry detection of the bacteria has been further integrated with a smartphone as a point-of-care (POC) portable platform to detect *E. coli* with high sensitivity [99]. Positively charged functionalized AuNPs [(+)-AuNPs] can interact with negatively charged bacteria. Due to the electrostatic interaction, the complex formation of (+)-AuNPs-bacteria could further interact with monoclonal antibodies, producing an intense color, as shown in Figure 2iv. A novel lateral flow strip to detect bacteria was developed following the mechanism [100].

Adding a linker to the NPs could induce significant signal differences while developing a sensitive detection system of pathogens. The system can separate and concentrate *E. coli* 0157:H7 from the aqueous sample by using AuNP-coated starch magnetic beads. A bifunctional linker, 4× gold-binding peptide-tagged Streptococcal protein G (4GS), was used in this development. The linker exhibited a significant and unique fingerprint signal during surface-enhanced Raman scattering (SERS) measurement [101]. Various other properties of NPs are also explored, such as SiO_2_ nanostructure to detect *E. coli* O157:H7 [102] for acoustic wave biosensor, AuNPs [103], or TiO_2_ nanoparticles [104] to monitor *S. typhimurium* infections for optical-based biosensors. The developed immunosensor was based on the photoluminescence property of TiO_2_ NPs, whereby the change in the photoluminescence signals can monitor the interaction between the antibody and *Salmonella* antigen. In addition, biosensors based on the loop-mediated isothermal amplification (LAMP) method with AuNPs was developed to monitor *Salmonella* spp. [105], *Streptococcus iniae* [106], etc. With the advancement in nanotechnology, certain limitations of organic dyes or fluorophores could be minimized, such as better photostability, minimal chemical degradation, etc. Determination of multiple targets is possible because the emission of different fluorescent signals depends on the nanoparticles’ size and composition (NPs).

Upconversion nanoparticles (UCNPs) are other interesting fluorophores that can emit visible radiation from the excitation at the near-infrared wavelength range due to the nonlinear optical principle. UCNPs may possess attractive optical and chemical characteristic features due to the anti-Stokes luminescence properties, auto-induced light scattering by biological samples, and no autofluorescence that significantly improve the signal-to-background noise ratio. The physical and chemical properties of natural materials are explored to develop such a biosensing system. The fluorescence quenching or recovery phenomenon due to the interaction of AuNPs-aptamers and UCNPs-cDNA in the presence of target bacteria was studied based on fluorescence resonance energy transfer (FRET). AuNPs as acceptors and UCNPs as donors were conjugated with aptamers and complementary DNA (cDNA), respectively. Due to the interaction and formation of the reaction complex [UCNPs-cDNA-AuNPs-aptamer], upconversion fluorescence quenching was observed. In the presence of targeted bacteria, dissociation of the reaction complex resulted in the recovery of upconversion fluorescence. The method was used in food and water samples analysis for tap water, pond water, and milk to detect *E. coli,* which could be detected within 20 min [107]. Such detection principles are also employed to detect other pathogenic bacteria, such as *Staphylococcus aureus* [108]. Instead of employing AuNPs, a tungsten disulfide (WS_2_) nanosheet was explored as an acceptor to develop a FRET-based aptasensor to detect *E. coli.* Strong upconversion fluorescence is quenched during the reaction of the UCNPs-aptamer-WS_2_ nanosheet due to 3D arrangement and possible van der Waals force interaction between the aptamer and the WS_2_ plane. However, the quenched fluorescence could be recovered because of the higher affinity of the specific aptamer with *E. coli* [109]. In order to simultaneously detect multiple pathogens, the structure–chemical relationship/interaction of natural materials and biosensing platforms or recognition elements can be investigated. UCNPs are further functionalized with the guanidine group (UCNPs@GDN), possessing positive charge and hydrogen donor sites. The negatively charged bacteria and UCNPs@GDN interact through electrostatic or hydrogen bonding interactions. Through this approach, multiple bacteria with seven different pathogenic bacteria, such as *E. coli, Salmonella, S. aureus, S. flexneri, C. sakazakii, L. monocytogenes,* and *V. parahaemolyticus,* and a total number of bacteria could be quantified [110]. Another approach to screening multiple pathogens in a complex matrix is the multicolor coding UCNPs. The multicolor coding may be achieved by adding different concentrations of sensitizer on the surface of the synthesized UCNPs. Sensitizer such as Yb^3+^ was used to dope UCNPs, and the doped UCNPs emitted red-green luminescence upon irradiation at the 980 nm (NIR) wavelength. Luminescence intensity varies due to different doping concentrations of Yb^3+^, and the five different foodborne pathogens of *E. coli* O157:H7, *S. paratyphi* B, *S. paratyphi* C, *S. enteriditis, and S. choleraesuis* could be distinguished by the red/green ratios obtained [111]. Combining upconversion nanocrystals codoped with Li^+^ and K^+^ increases the signal intensities by 7–10 times and can simultaneously perform a dual-target assay for *Y. pestis* and *B. pseudomallei* [112]. Another aspect is that the doping of UCNPs with different materials has changed the luminescence property; in this study, Mn^2+^, when doped to UCNPs (NaYF4:Yb, Tm), produced an intense peak at 807 nm. The developed system detected *S. typhimurium* [113]. ELISA is considered a gold standard for the immunoassay system. However, the method cannot reveal the early stage of infection. In this study, the authors developed an upconversion-linked immunosorbent assay (ULISA) by conjugating the upconversion NPs with streptavidin to detect the bacterium *M. plutonius*. The developed system could detect the bacteria as low as 340 CFU/mL, which is 400 times more sensitive than the standard ELISA method [114]. Additionally, UCNPs-based electro-driven immunochromatography assay (EICA) uses electroosmotic flow to enhance the sensitivity and reduce reaction time to detect pathogens such as *Y. pestis* EV76 [115].

Different carbon nanomaterials, such as carbon dots (CDs) [116], carbon nanotubes (CNTs) [117], graphene [118], and graphene oxide [119], have emerged as potential candidates to develop next-generation miniaturized biosensors due to their inherent physical, chemical, antimicrobial, and electrical properties. Carbon nanomaterials have been utilized to develop miniaturized diagnostic devices targeting pathogenic microbes following different detection principles, such as optical [120] and electrochemical methods [121]. Breakable organosilica nanocapsules (BONs) were employed to entrap and release CDs to detect *S. aureus* following fluorescence signals. The signal could be significantly amplified using the CDs@BONs strategy. The magnetic separation technique was also employed to increase the selectivity of the device [122]. Wang et al. synthesized positively charged nitrogen-rich carbon nanoparticles (pNC) to capture the target bacteria, i.e., *S. enteritidis*, and generate an optical signal. The pathogen and pNC interact through non-covalent interactions such as electrostatic and hydrogen bonding; the antibody further captures the complex at the test line, forming a pNC-bacteria–antibody sandwich complex. The novel lateral flow immunoassay system developed was label-free and straightforward. It exhibited excellent sensitivity, with LOD of 10^2^ CFU/mL [123].

Two or more nanomaterials may be incorporated into one system to produce significantly better results. The hybrid system could offer more advantageous properties than the individual materials. Recently, to detect food pathogens such as *E. coli*, an electrochemical immunosensor was developed where the electrode is composed of the hybrid nanocomposite of chitosan, MWCNT, PPy, and AuNPs. The authors have modified the pencil graphite electrode with a bionanocomposite of PPy/AuNP/MWCNT/Chi by drop-coating. Under optimum conditions, the modified immunoelectrochemical sensor could detect Gram-negative *E. coli* O157:H7 with a high selectivity of ~30 CFU/mL as its LOD [124]. Nanoparticles and graphene have gained massive attention due to their exciting chemical and physical properties. AuNPs were recently functionalized with 3D graphene to develop a DNA biosensor. In this study, the nanocomposite of 3D G-AuNPs enhanced the intrinsic properties of the materials to exhibit promising biosensing performance. With this nanocomposite material, the authors demonstrated electrochemically detecting dissimilatory sulfite reductase gene from sulfate-reducing bacteria. The DNA biosensor exhibited high sensitivity, with an LOD of 9.41 × 10^−15^ M of the target DNA [125]. Silver/graphene nanocomposites enhance the physical and chemical properties suitable for developing diagnostic devices with high conductivity and flexibility that individual materials could not provide. In another development, nanocomposites were prepared using a one-step laser-induction method where gold, silver, and platinum nanoparticles were uniformly distributed on the graphene surface. This method fabricated flexible impedimetric sensors and could detect *E. coli* O157:H7 as low as 1× 10^2^ CFU/mL with high specificity [126]. Magnetic nanoparticles (MNPs) and quantum dots (QDs) were combined to develop a portable optical biosensor system for *E. coli* O157:H7. In this double-layer channel system, MNPs acted as the channel to capture and concentrate the target bacteria. The QDs assisted in quantitatively analyzing the target analyte through its fluorescence signal. The biosensor offered LOD of 14 CFU/mL in 2 h [127]. Conventional biosensors are not widely known to distinguish between live and dead cells. Recently, an organic–inorganic hybrid nanoflower-based biosensor to detect live bacteria in a urine sample has been demonstrated. The organic component, such as GOx (glucose oxidase) and horseradish peroxidase (HRP), reacted with an inorganic component, such as Cu_3_ (PO4)_2_, to produce hybrid nanoflowers. The advantage of the hybrid nanoflower is the enhancement of the electrochemical signal and the detection selectivity of T4 phages [128].

Metal-organic framework (MOF) has many attractive features, such as large surface area, high porosity, and good stability. Such materials have broad application potential. Recently, colorimetric detection of pathogenic bacteria such as *S. aureus* was achieved with copper-MOF nanoparticles, which acted as peroxidase-like characters and catalyzed 3,3′,5,5′-tetramethylbenzidine in the presence of hydrogen peroxide. In this, Cu-MOF NPs were synthesized following the solvothermal method with copper nitrate and 2-aminoterephthalic acid as the starting materials [129].

### 4.2. Molecularly Imprinted Polymers (MIPs) Based Systems

**Figure 3:**
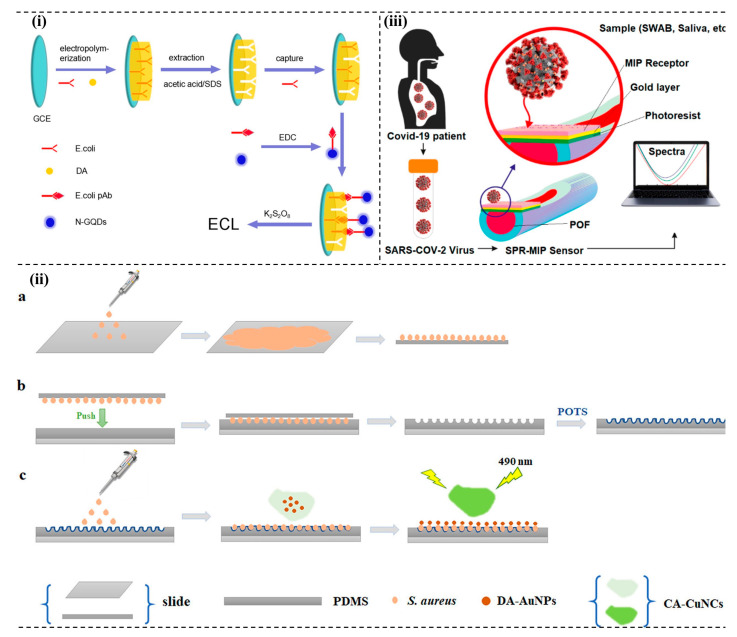
MIP-based pathogen detection system. (**i**) Fabrication schemes and detection of *E. coli* following ECL principles. Reprinted with permission from Ref. [134]. Copyright 2017 American Chemical Society. (**ii**) (**a**) bacteria template preparation and fabrication, (**b**) POTs-modified imprinted PDMS film, and (**c**) interaction of bacteria with the interface and FRET surface. Reprinted with permission from Ref. [137]. Copyright 2021 American Chemical Society. (**iii**) Outline of SARS-CoV-2 and plasmonic optical fibers (POF) sensors in different matrices [143].

MIPs, also called artificial receptors, are designed and synthesized to resemble the natural antibody–antigen system that can function like natural recognition elements [130]. Molecular imprinting technology allows for designing specific artificial receptors by following standard free radical polymerization methods or sol-gel processes [131]. Based on the interaction of functional monomers and target molecules, MIPs are of three types: covalent, semi-covalent, and non-covalent interactions. The system explores various detection principles, such as affinity-based sensors [132] and electrochemical methods [133]. Optical-based detection methods detect *E. coli* O157:H7 by employing the electrochemiluminescence (ECL) method. Figure 3i shows that surface imprinted polymer (SIP) could be achieved by electropolymerization of dopamine to detect *E. coli* O157:H7. In addition to that, nitrogen-doped graphene quantum dots are also employed along with potassium persulfate to produce an intense ECL signal. In optimum reaction conditions, the system could detect the bacteria as low as 8 CFU/mL and obtain linear relationships from 101 CFU/mL to 107 CFU/mL [134].

Quartz crystal microbalance (QCM) is a very sensitive analytical technique. The combination of MIP from polyurethane and QCM detects both *E. coli* bacteria and *B. subtilis* spores in the samples. The developed biomimetic system can study and monitor the growth and behavior of bacteria in different environments [135]. In another study, classical swine fever virus (CSFV) was detected using MIP as a receptor on a QCM-based sensor [136]. The MIP/QCM sensing platform also demonstrated rapid analysis that opens up quick testing for CSFV. Using *S. aureus* bacteria as a template, an imprinted polymer was designed on polydimethylsiloxane (PDMS), followed by further modification through the chemical vapor deposition method with the 1H,1H’,2H,2H’-perfluorooctyltriethoxysilane (POTS), as shown in Figure 3ii. The competitive interaction of bacteria with the FRET platform also increases fluorescence intensity [137].

MIP thin-film modified surface of screen-printed carbon electrodes (SPCE) have been used to enhance the interaction with the bacterial endotoxins and lipopolysaccharides (LPS) from *P. aeruginosa*. Sol-gel methods produced LPS-MIP active surfaces through the technique. Stoica and co-workers demonstrated that, to increase the adhesion of the LPS-MIP film on the substrates, two more silane monomers, 3-(2-trimethoxysilyl)-propyl methacrylate and tetraethyl orthosilicate, in the precursor solution could be used. Through this study, the developed LPS-MIP-SPCE interface recognizes LPS from *P. aeruginosa* more than LPS from *E. coli* [138]. In another study, organosiloxane polymers were used for imprinting *E. coli* to develop fast, sensitive, and affordable biosensors. The developed *E. coli*-imprinted organosiloxane polymers with polyethylene glycol (PEG) are 4.5 times more selective than other polymers, such as polydimethylsiloxane and organosiloxane without PEG [139].

Detection of *L. monocytogenes* is also possible using the MIP technique. The developed method does not require pretreatment of the sample and offers effective detection of *L. monocytogenes* in food samples [140]. Integrating MIP techniques with surface plasmon resonance (SPR) enhances the detection performance. Sensor chips of SPR were prepared using MIPs to detect RoxP, a protein with high antioxidant activity. The LOD and dissociation constant discerned for the protein were 0.23 nM and 3.3 × 10^−9^ M, respectively [141]. An SPR biosensor for *S. aureus* α-hemolysin was also developed by modifying the SPR sensor with MIPs to enhance its sensitivity and affinity (K_D_ = 2.75 × 10^−7^ M) for the pathogenic microbe [142]. A plasmonic sensor was also developed to detect severe acute respiratory syndrome coronavirus 2 (SARS-CoV-2) with MIPs, as shown in Figure 3iii. The developed sensor is sensitive and faster than the standard RT-PCR-based detection (10 min compared to 3–4 h) [143].

### 4.3. Hydrogels Based Systems

Hydrogels are polymers that swell with water and retain water molecules inside their hydrophilic polymer matrix. They possess features such as a 3D network, porous structure, etc., that enhance their application in advanced technology. Hydrophilic monomer, initiator, and crosslinker are integral in hydrogel preparation through copolymerization or crosslinking processes [144]. Hydrogels can provide a natural-like microenvironment for biomolecules that prolong the biochemical activity. The hydrogels find application in detecting microbial pathogens following different strategies [145]. Recently, a gelatin-based photonic hydrogel sensor for *P. aeruginosa* was developed. In the presence of the target pathogen, the hydrogel expands due to crosslinking and lattice spacing disruption, resulting in the redshift of lights. Thus, the presence of a pathogen could be identified visually due to the change in wavelength of the photonic hydrogel [146]. In another development, a highly sensitive T2 biosensor (i.e., direct transverse relaxation time biosensor) for foodborne pathogens such as *S. enteritidis* was developed to detect 50 CFU/mL of the microorganism within two hours. In this study, the hydrogel’s sol-gel property and the enzymes’ high selectivity were explored. Instead of focusing on the magnetic property as in conventional methods, the study focused on manipulating the relaxation behavior of water molecules using alkaline phosphatase (ALP) mediated sol-gel transition of hydrogel without compromising the sensitivity of the detection method [147].

**Figure 4:**
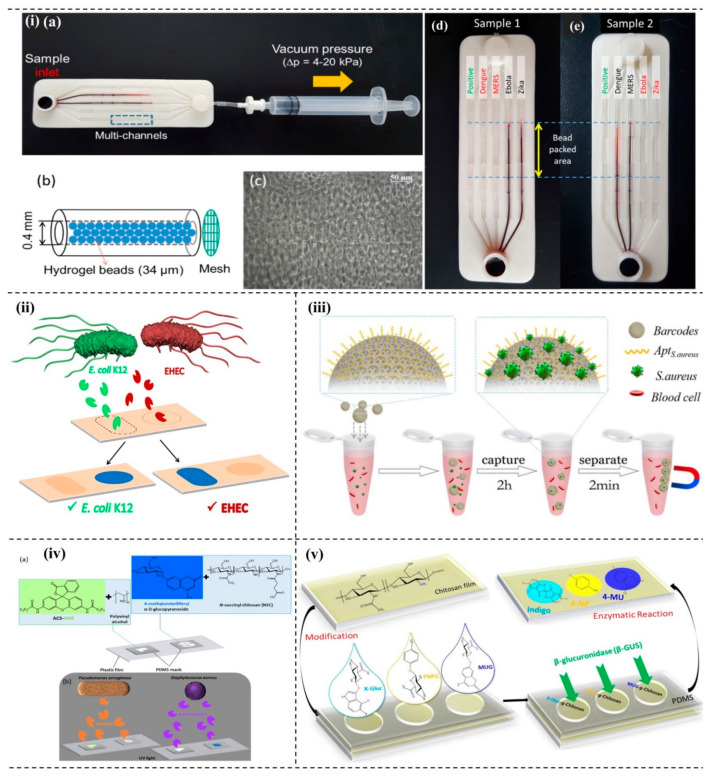
Hydrogel-based pathogen detection system. (**i**) Biosensor system using DNA hydrogels: (**a**) photograph of the kit, (**b**) bead—packed microchannel, (**c**) microscopic image of bead—packed microchannel, (**d**) sample 1-Dengue and MERS, and (**e**) sample 2–Ebola and Zika [148]. (**ii**) Selective detection system of pathogenic and nonpathogenic bacteria-selective discrimination of *E. coli* K12 and EHEC. Reprinted with permission from Ref. [149]. Copyright 2018 American Chemical Society. (**iii**) Aptamer-based hydrogel barcodes to capture and detect bacteria. Reprinted with permission from Ref. [150]. Copyright 2018 Elsevier. (**iv**) Hydrogel assisted detection system of elastase and α-glucosidase: (**a**) chemical structures of substrates and matrices, (**b**) the fluorescence output of the shape-encoded letters under UV light. Reprinted with permission from Ref. [151]. Copyright 2020 American Chemical Society. (**v**) Selective detection of bacteria using chitosan hydrogel-fabrication and investigation of the reaction on PDMS chip. Reprinted with permission from Ref. [152]. Copyright 2018 John Wiley and Sons.

A microfluidic biosensor system can quickly detect multiple viruses on a single platform. A critical addition in developing the plan is the incorporation of DNA hydrogels on the surface of the microbeads by the rolling circle amplification (RCA) process, as shown in Figure 4i. The process depends on the rapid formation of DNA hydrogel on each microbead surface in the microfluidic channel. In this technique, if the target pathogen comes in contact with the corresponding template, rapid amplification occurs through RCA processes that increase the surface area of the microbead. The integration of multi-channel microfluidics assisted in detecting many viruses, such as Zika, Ebola, MERS, and dengue, within 15 min [148]. Differentiation among bacteria is vital to discriminate the toxic and non-toxic pathogens selectively. A hydrogel-assisted discrimination technique of the enterohemorrhagic *E. coli* (EHEC), a food-borne highly toxic pathogen, from a non-virulent *E. coli* K12 was reported (Figure 4ii) [149]. Clinically, identifying bacteria takes 3–5 days of incubation and more than 10 h to grow on the solid platform. In a recent study, newly developed aptamer-based barcode technology could capture and detect bacteria simultaneously within 2.5 h in a complex sample (Figure 4iii). The poly (ethylene glycol) (PEG) hydrogel inverse opal particles make up the barcodes and possess a unique reflection peak. Because PEG hydrogel shows anti-adhesion, it reduces non-specific binding significantly, increasing the selectivity and sensitivity of the developed system. The developed aptamer-based hydrogel barcode could effectively detect bacteria as low as 100 CFU/mL [150]. Bacteria produce characteristic enzymes; for example, *P. aeruginosa* and *S. aureus* produce elastase and α-glucosidase, respectively. The bacterial species could be indirectly detected using a shape or probe encoded hydrogel on the sensing surface by detecting elastase and α-glucosidase. Figure 4iv shows the fluorescence output with letters P (green) and S (blue) as examples. In 60 min observation time, the developed system could detect as low as ≤ 20 nM and ≤ 30 nM of elastase and α-glucosidase, respectively [151]. β-glucuronidase (β-GUS) is secreted by more than 98% of *E. coli* strains. To reduce false-positive results, three different chromogenic and fluorogenic substrates were incorporated in chitosan hydrogels to detect the characteristic enzyme of the bacteria. The three chromogenic substrates used in the developed system are 4-nitrophenyl-β-D-glucuronide, 5-Bromo-4-chloro-3-indolyl-β-glucuronide, and 4-methylumbelliferyl-β-D-glucuronide (Figure 4v); the naked eye could visualize the released dye within 80 min, whereas spectroscopically, the detection could be done within less than 60 min [152]. The researchers have further developed a hydrogel that functionalizes the three different chromogenic enzyme substrates of α-glucosidase, β-galactosidase, and β-glucuronidase, and the LOD of the system with the respective substrates was 0.2, 3.4, and 4.5 nM. Different strains secreted different enzymes, so the interaction and colorimetric detection of the specific bacterial strains with the chromogenic substrates could be visualized on a conventional microplate reader [153].

Hydrogel-based detection of SARS-CoV-2 has been reported where a highly sensitive microfluidic-based biosensor using the RCA technique was used. The authors explored the rolling circle amplification of the pathogen DNA and immobilized probe that formed DNA hydrogel. Upon optimization, it could detect the virus as low as ~ 30 aM in 5 min. The developed method is ultrasensitive and can be used in POCT without any sophisticated device [154]. Different materials are also incorporated to prepare composite hydrogels to enhance its applications and robustness. Hydroxypropyl chitin/tannic acid/ferric ion (HPCH/TA/Fe) were used to develop a simple assembly composite hydrogel, which is thermosensitive and pH-sensitive [155].

### 4.4. Photonic Crystal Based Systems

Photonic crystals (PCs), also called photonic band-gap materials, are suitable for optical sensing applications because the flow of light through these materials can be controlled and manipulated [156]. The periodic arrangement of atoms or molecules in the crystal lattice structure and the periodic dielectric structure of the PCs produce band gaps that uniquely interact with light. To obtain various orientations and 1D, 2D, and 3D structures of PCs, one can follow lithography, self-assembly, stacking, and other techniques to incorporate microcavities, waveguides, and porous geometries, etc. [157]. PCs in biosensors allow the detection of various pathogenic microbes. In a recent development, 1D PC platforms have been fabricated to determine bacterial contaminants such as *E. coli.* The developed colorimetric system focused on easier fabrication and lower-cost processes to design novel hybrid plasmonic–photonic devices by incorporating dielectric and electro-optical responsive plasmonic materials. Such design produces a sensitive device that can sense even a slight alteration in the surrounding environment due to specific interactions of the metals [158]. 1D photonic crystal biosensors can detect *E. coli* [159]. By incorporating 2D PCs, the waveguide biosensor can detect the DH5α strains of *E. coli* [160]. A gelatin-based photonic hydrogel sensor to detect *P. aeruginosa* was also proposed. The system has the potential to distinguish various bacteria, such as *E. coli*. In the presence of *P. aeruginosa*, red-shifts reflection spectra occurred due to the expansion of hydrogel, which in turn increases the lattice spacing [146]. Liu and co-workers explored the photonic crystal-based biochips to detect bacteria in urine samples with the help of a machine vision (MV) diagnostic system [161]. The MV algorithm gives precise results, and apparently is a promising technology for developing POC applications. The antibody-modified nanoparticles and target biomolecules (bacteria) interact through the antibody–antigen interaction principle. The MV algorithms enable the digital signals of the emitted luminescence to process, resulting in easy and quick quantitative analysis that is possible due to the integration of photonic crystals into the biochip system.

### 4.5. Ionic Liquids (ILs) Based Systems

Recently, biosensing technology has incorporated ILs because of their attractive physical and chemical properties, and they can be engineered for various applications, including the design of stimuli-responsive materials. ILs are organic chemical compounds with cations and anions or organic salts, due to which they possess unique properties [162]. In a recent study, researchers investigated magnetic ionic liquids (MILs) to enrich and preconcentrate the pathogens from an aqueous solution. Through this strategy, the preconcentration of *E. coli* could achieve an enrichment factor of 44.6 within 10 min, and a LOD of 10^2^ CFU/mL could be achieved [163]. The work was extended to study the detection of enriched *Salmonella*. The integration approach of MIL and recombinase polymerase amplification (MIL-RPA) enabled LOD of 10^3^ CFU/mL of *Salmonella. T*hese studies demonstrated that the MILs-based strategy is an effective route for enrichment and extraction of the target pathogenic microorganisms [164]. There have been gradual improvements in bacterial detection, considering all the major limitations. Magnetic beads (MBs) were used to concentrate the carriers in one such developmental stage. The functionalized MBs with specific recognition agents, such as antibodies or aptamers, increase the sensitivity. The magnetic property facilitated concentrating the pathogenic bacteria.

### 4.6. Responsive Polymer Based System

**Figure 5:**
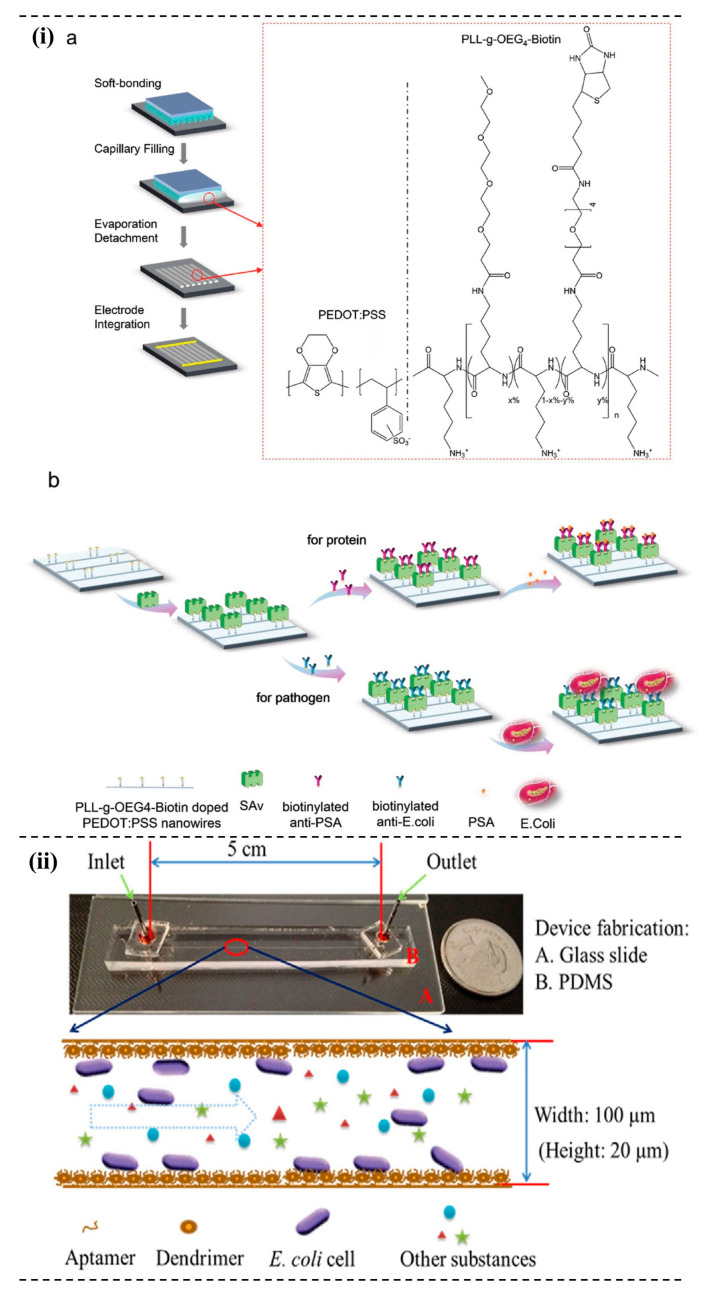
Responsive polymer-based pathogen detection system. (**i**) (**a**) Fabrication steps of the immunosensor using conductive polymers such as PEDOT:PSS, and (**b**) immobilization and detection strategies of *E. coli* using the nanoarray setup. Reprinted with permission from Ref. [167]. Copyright 2021 American Chemical Society. (**ii**) PDMS dendrimer-aptamer-RCA detection system in which PAMAM dendrimers are used to decorate the microchannels that enhances the *E. coli* detection 50 times. Reprinted with permission from Ref. [168]. Copyright 2017 Elsevier.

Responsive polymers respond to various stimuli such as light, temperature, pH, pressure, electric/magnetic field, force, etc. [165]. Currently, polymers responding to a change in temperature is the most studied and understood. Some polymers have a specific phase transition temperature called the lower critical solution temperature (LCST). Below LCST, polymers and solvent molecules are in one phase, i.e., homogeneous mixed-phase, whereas above LCST, phase separation happens. Poly (N-isopropyl acrylamide) (PNIPAM) is one of the most extensively studied polymers that exhibit the LCST phenomenon. The mechanistic understanding of the PNIPAM-based system that exhibits the LCST phenomenon is that the polymer is soluble below 32 °C and precipitates above 32 °C in an aqueous solution. Water molecules and amide functional groups in polymers could interact predominantly through the hydrogen bonding below LCST. In contrast, above the LCST, the polymer cannot retain water molecules due to the breaking of hydrogen bonding between them [166]. By copolymerizing with chemical compounds or monomers, one can control the property of the polymer by controlling the polymer composition and architecture. Xue and co-workers demonstrated the printing of uniform nanowires array using nanoscale-printing technology. Commercially available conductive polymers such as poly (3, 4-ethylenedioxythiophene)-poly (styrenesulfonate) (PEDOT:PSS) are doped with PEGlated biotin-derivatized polyelectrolytes and printed on the nanowire surface, as shown in Figure 5i. This highly ordered nano-array setup could detect *E. coli* as low as 10 CFU/mL [167]. Rolling circle amplification (RCA) is a powerful method for DNA amplification, and the authors employed the technique to enhance the detection sensitivity of *E. coli* O157:H7 in the microfluidic system. In the system, poly (amidoamine) (PAMAM) dendrimer decorates the microchannel, as shown in the Figure 5ii, immobilized with aptamers. By incorporating the RCA techniques, the signal could be enhanced 50 times and detected as low as 10^2^ CFU/mL [168].

As stated above, detection systems for infectious pathogens have started exploring the potential application of smart materials to develop robust and high throughput technology. A brief summary of the emerging smart materials for microbial pathogens is shown in Table 3.

**Table 3:** Emerging smart materials as pathogen detection systems.

Detection Systems	Advantages	Disadvantages	LOD	Ref
Nanomaterials based systems	(i) High specific surface area (ii) Sensitivity of the system may increase (iii) Less sample volume is required (iv) Hybrid nanomaterials may exhibit better performance (v) Strong amplification of signals	(i) Immobilization of bio molecules on it is a challenge (ii) Cytoxicity and toxicity effect of many metal and metal oxides nanomaterials are reported (iii) Nanomaterials modified antibodies are expensive	50 CFU/mL	[97]
			10.7 CFU/mL	[108]
			340 CFU/mL	[114]
			10^2^ CFU/mL	[169]
			30 CFU/mL	[124]
MIP based systems	(i) Highly sensitive and specific (ii) MIPs are very stable and cost-effective (iii) Good reproducibility (iv) Capable to tailor the recognition site for target molecules	(i) Less selective as compared to natural enzymes (ii) All molecules cannot be imprinted (iii) Time consuming to design and synthesis MIP (iv) Tedious characterization	8 CFU/mL	[134]
			1.7 µg/mL	[136]
			11.12 CFU/mL	[137]
Hydrogel based systems	(i) Possess high degree of flexibility (ii) Biocompatible (iii) Hydrogels can be injected and easy to modify	(i) Low thermal resistance (ii) Non-adherent (iii) Low mechanical strength (iv) Difficulty in handling and loading	50 CFU/mL	[147]
			100 CFU/mL	[150]
			~3 aM in 15 min and 30 aM in 5 min	[154]
Photonic Crystal based system	(i) Highly sensitive (ii) Fabrication does not require clean room facility (iii) Short assay time (iv) Wide detection array (v) Relatively large bandwidth	(i) Challenges in constructing 3D scale (ii) Limited to specific frequencies (iii) Scattering loss at air–glass interfaces (iv) Tunability of the slowdown factor in given structure	174a nm/RIU	[159]
			Not mentioned	[160]
			Not mentioned	[146]
			Not mentioned	[161]
Ionic Liquid based systems	(i) Both conductor and binder (ii) Good catalytic ability and super sensitivity (iii) High thermal stability	(i) Relatively expensive as compared to conventional organic solvents (ii) High cytotoxicity (iii) Mostly limited to electro-analytical system	10^2^ CFU/mL	[163]
			10^3^ CFU/mL	[164]
Responsive Polymer based system	(i) Multifunctionality (ii) Structural stability (iii) Facile integration in the detection devices (iv) Tunable detection sensitivity	(i) Tedious synthesis process of the designed responsive polymer (ii) Lack of toxicity data profile	10 CFU/mL	[167]
			10^2^ CFU/mL	[168]

## 5. New Perspectives and Emerging Pathogenic Detection Devices

Rapid, reliable, and low-cost portable detection systems for various disease-causing agents, environmental pollutants, and health indicators are in great demand in civilized societies. Researchers made significant progress in this interdisciplinary technological field on different scientific fronts, such as material sciences, microscale technologies, computer sciences, electronics and telecommunications, and biological sciences [170]. Sustainable concepts such as a lab-on-chip, single-use POC devices, self-powered biosensors, and, of late, smart materials have caught the eye of the research community [171]. The future of pathogen detection is the portable detection devices along with wearable/implantable biosensors, hybrid pathogenic sensors, and multiple assay sensors. The implementation of micro- and nano-scale innovative technologies has enabled biosensors to be more versatile, robust, and dynamic. Miniaturization is the fundamental requirement in constructing biosensors, particularly POC devices. In this section, we will briefly assess the development of pathogen detection using emerging techniques and approaches, such as droplet-microfluidic systems, paper-based systems, smartphone-based systems, multiple assay devices, and wearable biosensors, since 2016 onwards.

### 5.1. Droplet Microfluidic System

Droplet microfluidics have at least two phasic environments with dispersed and continuous phases to form highly dispersed microdroplets. In this field, the droplets can be studied either discretely on the electrodes or in the closed microchannels. Inside the microchannel, the fundamental principle of droplet formation is the outcome of the interfacial tension between the phases to reduce the interfacial area [172]. The advantage of employing such technology is the possibility to perform chemical or biochemical reaction studies simultaneously in a massive number of droplets. The droplet functions as a micro-reaction chamber and fluid transportation unit [173]. Due to various advantages, the technique has been explored for the detection of disease biomarkers and pathogens in crude samples. For diagnostic application, nucleic acid amplification such as PCR has been employed, especially in bacterial or viral outbreaks. However, due to the limitation of conventional PCR instrumentation and complex procedures, droplet microfluidics is a promising alternative solution. The technique, along with the loop-mediated isothermal amplification (LAMP) method, is employed to develop a sensitive biosensor for *Salmonella typhimurium*. In comparison with the standard conventional methods, the developed method of LAMP-assisted droplet microfluidic technology is fast, specific, sensitive, and simple to operate. [174]. Multiple pathogens, such as *Bacillus subtilis, Legionella pneumohila, Vibrio parahemolyticus,* and *Listeria monocytogenes,* are also detected using the approach, with LOD 500 times lower than the conventional bulk-phase LAMP method [175]. Droplet microfluidics also enable the study down to a single cell, as the cell can be encapsulated inside the droplets. *Salmonella* at the single-cell level could be specifically detected by employing a novel microdroplet approach [176]. In addition to these, microfluidic droplet technology has the potential to do surveillance for infectious pathogens such as bacterial in fresh-cut wash water [177], a virus such as SARS-CoV-2 [178], and automated detection and monitoring of chemical and biological warfare agents [179].

### 5.2. Paper-Based System

Paper as a sensing platform has been explored because it offers several physical, chemical, and biological advantages over many existing sensor platform materials. Some of the advantages are wicking property for passive fluid flow through microfluidic channels, availability, biocompatibility, easy surface functionalization, lightweight and flexibility to design portable devices, white color background for a better colorimetric response, etc. Paper-based devices such as dipstick and lateral flow assay-based optical detection sensors are common. The device has been explored to monitor and detect various pathogenic microbes, such as *E. coli* [180], *C. albicans* [181], *Neisseria meningitides* [182], *Salmonella* [183], *S. typhimurium* [184], *Cronobacter* spp. [185], and other bacteria [186].

**Figure 6:**
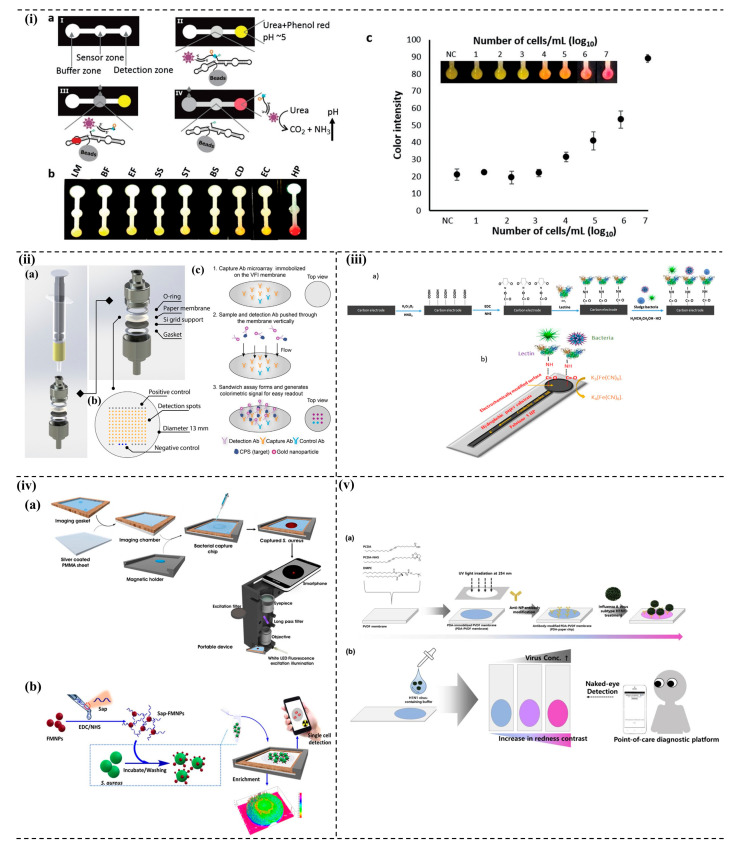
Emerging detection approach for pathogen. (**i**) (**a**) Colorimetric detection of *H. pylori* using paper-based microfluidic device, (**b**,**c**) selectivity and sensitivity of the developed device. Reprinted with permission Ref. [187]. Copyright 2019 John Wiley and Sons. (**ii**) Vertical flow immunoassay (VFI) system to detect *B. pseudomallei*: (**a**) VFI platform and layers, (**b**) microarray design, and (**c**) operation workflow. Reprinted with permission from Ref. [189]. Copyright 2019 Elsevier. (**iii**) Impedimetric paper-based biosensor for bacteria: (**a**) surface modification of electrode surface and detection principle, and (**b**) functionalized screen-printed probe for bacteria detection. Reprinted with permission from Ref. [197]. Copyright 2018 Elsevier. (**iv**) Smartphone-based biosensor for *S. aureus* detection: (**a**) construction of sealed chamber and the bacterial detection cassette, and (**b**) detection steps of pathogen and quantification using smartphone. Reprinted with permission from Ref. [207]. Copyright 2018 Elsevier. (**v**) Polydiacetylene-based paper chip and colorimetric detection of pH1N1 virus: (**a**) fabrication and preparation of paper-chip, and (**b**) colorimetric detection of pH1N1 virus. Reprinted with permission from Ref. [208]. Copyright 2019 Elsevier.

A simple and sensitive paper-based device was developed to detect *Helicobacter pylori* (*H. pylori*). The authors designed a sensor molecule based on RNA-cleaving DNAzymes. The schematic of the paper-based device is shown in Figure 6i. The advantages of the device may be attributed to the simple sample processing from human stools, stability for four months at room temperature, and colorimetric read-out within minutes [187]. In order to increase the portability and simple-to-use operation, Fu and co-workers integrated a paper-based chip with nucleic acid extraction and amplification. The developed device can detect *L. monocytogenes* as low as 10^4^ CFU/mL and offers advantages such as reducing operation steps, cross-contamination prevention, and efficient detection [188]. Figure 6ii shows a paper-based vertical flow immunoassay (VFI) that utilizes the nanoporous nitrocellulose membrane to detect biothreat pathogens. One of the limitations of lateral flow immunoassay using nitrocellulose membranes is the pore size, which affects the flow rate and the assay sensitivity. Though the developed approach is also membrane-based, the fluidic movement is vertical in VFI, instead of parallel. Through this approach, a faster flow rate and narrower pore size improves the assay’s sensitivity by five times. The developed device detected *B. pseudomallei* and *B. anthracis* [189]. The paper-based analytical device can now detect bacteria and chemical metabolites such as nitrite with the same device. The newly designed PAD can detect 10^4^–10^7^ CFU/mL in 6 h of the bacterial concentration, *E. coli* to be specific, while studying UTI, whereas it can detect nitrite in the range of 0–1.6 mg/dL. Such multifunctional PADs would be cost-effective for application in POC and resource-limited settings [190].

Electrochemical platforms are the most commonly employed sensor platform for detecting pathogens [191]. The advances in electronics and the availability of microelectronic circuit designs that can be fabricated with simple techniques have made the electrochemical platform an ideal portable device method [192]. Integration of microfabrication techniques can lead to developing a device for POC applications. Current technologies can generate circuits of a few millimeter dimensions, enabling miniaturization, which significantly aids the generation of POC technologies. These developments have made it possible to carve in sensor platforms such as optical [119], FET [193], capacitive [194], potentiometric [195], among others. Khan et al. recently demonstrated the development of an electrically receptive and thermally responsive sensing platform by integrating graphene-PNIPAM-Au on the paper substrate to detect bacterial cells. The developed sensor produced ultrasensitive (10^1^–10^5^ CFU/mL) and highly reproducible (85–97%) results [196]. Many researchers focus on simple, low-cost, and portable devices. Recently, an impedimetric paper-based biosensor for bacteria in water was developed. Figure 6iii shows the reaction scheme, including the functionalization of the electrode surface and the detection. The developed system detected bacterial concentrations ranging from 10^3^ to 10^6^ CFU/mL and LOD of 1.9 × 10^3^ CFU/mL [197]. The electrochemical paper-based device (e-PAD) is now a prominent methodology for developing portable and affordable biosensors. One of the major highlights of the electrochemical-based biosensors is the sensitivity that can detect as low as nano- or femtomolar concentration and various pathogens, including *P. aeruginosa*, *S. aureus,* etc. Channon and co-workers integrated Au nanowires on the paper surface to enhance the detection and improve the paper-based electrochemical device. The authors were able to increase the signal order of magnitude in the detection limit when comparing it with the static paper-based biosensor. The LOD for West Nile Virus is 10.2 particles in 50 µL [198].

### 5.3. Smartphone-Based System

Integrating smartphones with portable sensors is a rapidly growing R&D activity in remote sensing, point-of-need (PON) monitoring, and healthcare (including POC and self-monitoring) systems [199]. The integration finds immense applications in the real-time monitoring of the analytes [200]. The wireless technology has dramatically boosted the sensing applications. These integrated hybrid devices are also rapidly infusing into the environmental sector for monitoring pathogens and toxicity in samples.

Recently, there have been many developments of smartphone-based biosensors to detect various pathogens, such as *S. typhimurium* [201], *S. enteriditis* [202], *E. coli* [203], COVID-19 [204], etc. The digital camera integration with a smartphone enables optical sensors to be practical. A smartphone-based malaria detection sensor apparatus fabricated on a multi-channel optic fiber was reported with a LOD of 264 pM. The optic fiber was sputtered with gold, followed by the functionalization of aptamers. The images of the light spots were captured from a smartphone and were further processed with image processing applications such as image-J [205]. Barnes et al. developed a smartphone-based LAMP system to identify pathogens, especially in urinary sepsis patients. The development has shortened the time of bacterial analysis compared to the standard clinical analysis methodologies (~1 h vs. 18–28 h). The device was effective against many Gram-negative and -positive pathogens and was cost-effective, and it has the potential to rapidly diagnose UTI and urinary sepsis. Additionally, it is configurable for multiple pathogens [206]. Shrivastava et al. developed a smartphone-based detection device to detect *S. aureus.* In the study, they used aptamer that was functionalized with fluorescent magnetic nanoparticles. Under optimal conditions, the device could detect as low as 10 CFU/mL of *S. aureus* within 10 min in a peanut milk sample. Figure 6iv depicts the fabrication and design of the device with the reaction steps, fluorescence image capturing, and processing [207]. In another development, Son and co-workers developed visual detection of pH1N1 virus on a polydiacetylene (PDA)-based paper sensing platform. The authors developed a smartphone app (virus detection) to detect the virus using the fabricated hybrid chip. Figure 6v shows the fabrication and development of the PDA-paper chip and the colorimetric detection of pH1N1 virus [208]. The device was compared with the commercial diagnostic kits; the sensitivity of the kits was comparable, which would significantly influence the smart-healthcare system.

### 5.4. Multiple Assay Devices (MADs)

One-stop analysis of multiple analytes is beneficial compared to single analyte assay systems. With the help of the multiple assay devices (MADs), the cost and time needed for the analysis could be significantly reduced. Most of the MADs currently available have huge setups with high costs. Some commonly used MADs are mass-spectrometer, multiplex PCR, next generation sequencing technologies [209]. Scaling down the size of the MAD systems is one of the research focuses of current analytical sciences. Electrochemical sensing is the most promising for MADs because of its high sensitivity and advancements in techniques, as screen printing has dramatically reduced the cost factor. A recently developed device detects multiple viruses, such as human coronavirus (HCoV) and Middle East respiratory syndrome coronavirus (MERS-CoV). An electrochemical sensor with eight carbon electrodes deposited with AuNPs was reported. Cysteamine and glutaraldehyde were immobilized on the electrode surface to adhere to HCoV and MERS. Blocking was performed with BSA to avoid any non-specific binding. The sensing method followed competitive antigen binding with the free virus in the samples in a given antibody concentration. This wearable sensor has a LOD of 0.4 and 1 pg/mL for HCoV and MERS, respectively [210]. In another development, Shin et al. developed a multiplexed detection of *E. Coli* O157:H7, *S. aureus*, *S. typhimurium*, and *B. cereus*. The handheld lateral flow assay device showed that it could detect bacteria from contaminated lettuce [211]. Recombinase polymerase amplification (RPA) paper chip biosensors were also developed to detect multiple pathogens such as *E. coli*, *S. typhimurium*, and *S. aureus*. The paper-based chip could detect the target pathogens as low as 10^2^ CFU/mL [212]. Another POC development to detect *L. monocytogenes* and *S. enterica* was based on surface-enhanced Raman scattering (SERS)-lateral flow (LF) combined with RPA methods. Under optimum conditions, the developed POC could detect as low as 27 CFU/mL and 19 CFU/mL for *S. Enteriditis* and *L. monocytogenes*, respectively [213]. Xiaofeng Wei et al. developed an instrument-free multiple aptasensor to detect three major food pathogens, i.e., *S. enterica, E. coli,* and *L. monocytogenes,* using a bar-chart microfluidic chip [147]. The developed spin-chip could detect as low as 10 CFU/mL and has the potential for quick detection of multiple pathogens.

Alternatively, MADs can also be developed to target a single pathogen but with multiple antigens specific for the same. This strategy can be used to eliminate false-positive results and increases the specificity of the biosensor. Colorimetric paper-based detection of two malaria biomarkers, i.e., *Plasmodium* lactate dehydrogenase (pLDH) and *Plasmodium falciparum* glutamate dehydrogenase (*Pf* GDH), with aptamers as biorecognition elements was developed recently in our group. Specific aptamers were used to capture the enzyme biomarkers. A substrate-dependent reaction quantified the captured *Pf-*LDH and *Pf*-GDH through a dye (resazurin) coupled colorimetric assay. It may be noted that PFLDH is a pan malaria-specific biomarker, and *Pf-*GDH is specific for *P. falciparum* infections [214].

Advanced sensing techniques like electrochemiluminescence (ECL) [215] are promising for developing MADs. The ECL is based on luminescence reactions at a specific redox potential for a particular donor–acceptor pair [216]. This property of ECL can overcome the current drawback, such as interference in analyzing multiple analytes. Most other sensor techniques have interference issues when multiple analytes are simultaneously probed on a single platform. Integrating nanomaterials and microfluidics on paper-based devices is also a current research interest for developing MADs. The next big step will be integrating MADs with the IoT (internet of things), enabling rapid diagnosis in real time.

### 5.5. Wearable Biosensors

Wearable biosensors (WBs) are expected to offer real-time information on specific biomarkers for assessing an individual’s health [217]. However, the quantification of biomarkers through WBs remains a challenge. It is generally composed of sensors coupled with short-range wireless technology such as Bluetooth, radio frequency identification (RFID), near field communication (NFC), etc., to transfer the sensor data to mobile devices such as a smartphone [218]. Information on the application of WBs to detect pathogenic microorganisms is limited. Ciui et al. developed a glove-based sensor to screen *P. aeruginosa*’s virulence factor. Conductive inks were printed on the index and middle fingers of the gloves to screen the targets. Under optimal conditions, the sensor could detect analytes such as pyocyanin and pyoverdine as low as 3.33 nM and 1.66 µM, respectively [219]. Nguyen et al. developed wearable materials that detect metabolites, chemicals, and pathogens. With these materials, a wearable mask was developed for the non-invasive detection of SARS-CoV-2 within a detection time of one and half hours and at ambient room temperature [220]. Wearable technology coupled with the IoT may help screen patients remotely, which may be suitable for monitoring and managing highly infectious pandemic diseases such as COVID-19 in community settings [221]. One of the limitations of the WBs is the battery or power supply. In the meantime, research on self-powered biosensors have been going on that can be explored to develop wearable technology to detect pathogenic microbes [222].

## 6. Conclusions

The threat from pathogenic microorganisms to human and animal health is well known. However, there is an increasing concern about the emergence of more deadly pathogens in environmental samples. Timely detection of the pathogens would greatly alleviate the burden in the healthcare and medical sectors and save lives. Over the last decade, research on developing pathogen detection has shifted from conventional lab-based analytical techniques to portable, low-cost, and reliable devices for rapid and large-scale processing of environmental samples. This research transition is clearly visible in this review work. The emergence of advanced materials (including smart materials), techniques (e.g., microfluidics, MEMS), and low-cost platforms (e.g., paper, PDMS) have significantly boosted the transition. This movement has been further emboldened by modern electronic communication (e.g., IoT) and technologies (e.g., smartphone) to expand the application horizon of portable devices, particularly for their applications in POC, remote-inaccessible locations, and personalized healthcare systems (including wearable devices). There has been a parallel effort to develop multiplexing and robust high throughput analysis suitable for rapidly processing large samples in community settings under endemic and pandemic (e.g., caused by SARS-CoV-2) situations. Technologies such as next-generation sequencing and microarrays have boosted these efforts. There are, however, many challenges to meet in order to develop sensitive, specific, stable, and low-cost portable detection devices for commercial applications. Generation and amplification of specific signals from the interaction between receptor (or bioreceptor) and the target pathogen are vital to impart sensitivity and specificity to the detection device. The volume of the current research on material sciences and signal transduction platforms is encouraging, giving hope of achieving many custom-made devices for sensitive detection of pathogens with high selectivity. There has been intensive parallel research over the last few years to improve the stability of the recognition systems and the devices. Efforts are on to improve the stability of the proteinaceous biorecognition elements (enzyme and antibody) and replace these elements with more stable molecules such as nucleic acid aptamer as an alternative means. The challenge of developing a stable and selective biorecognition system is also posed by rapid mutations in many pathogens that are likely to impair the detection strategies and selectivity of the devices. More intensive research is warranted to overcome these challenges and drawbacks for developing rapid, reliable, low-cost, and portable pathogen detection devices for real-world applications.

## Figures and Tables

**Table 1:** Advantages and limitations of conventional techniques used to detect microbial pathogens in environmental samples.

Technique	Advantages	Limitations
Culture-dependent Methods		
Multiple Tube Fermentation [25]	(i) Sensitive and sustainable (ii) Flexible sample volume (iii) Applicable to all kinds of water samples (iv) Broad indicators and alternative metrics (v) Easy to perform (vi) Low-cost media	(i) Less precision (ii) Large population of bacterial species can affect the detection (iii) Blockers can reduce the growth of species (iv) Difficult to track slow-growing or VBNC bacteria
Membrane Filter [25]	(i) Simple, and convenient (ii) Consistent results if the number of colonies are grown sufficiently (iii) Discrimination and recognition in the media (iv) Detection of small numbers of bacteria populations possible (v) Sometimes not demanded more cultivating steps	(i) Frequent variations in adsorption affect the growth of target organism (ii) Less selectivity (iii) Difficult to detect growth in turbid sample (iv) Time-consuming
Microscopic examination		
Microscopic examination [25]	Easy, fast and direct Inexpensive method Possible to perform routinely in a variety of clinical settings	Unable to identify large proportion of the microbial community Less sensitive than culture

**Table 2:** Advantages and limitations of advanced techniques used for the detection of microbial pathogens in environmental samples.

Technique	Advantages	Limitations
Immunological Methods		
Enzyme linked immunosorbent assay [60]	(i) Qualitative and quantitative methods (ii) Robust, flexible, simple to perform, and sensitive test (iii) Specific for the target organisms	(i) Cross-reaction of antibodies (ii) Require pre-enrichment step (iii) More vulnerable (iv) Restricted application for high-untargeted microbe levels (v) No differentiation between viable and non-viable microorganisms is currently possible without pre-cultivation
Immunomagnetic Assay [25]	(i) Ease of application (ii) Separating and detecting bacteria simultaneously possible (iii) Low instrumentation needs (iv) Efficient system: better interaction with target molecules	(i) Difficult to separate complex phenotypes (ii) Antibodies coated magnetic particles are expensive (iii) Require relatively large volume of sample and reagent (iv) Possible interference in fluorescent signal output
Nucleic Acid-based Methods		
Fluorescence in situ hybridization [61]	(i) Quick, sensitive, and safe test (ii) Consistent hybridization products (iii) Detection of VBNC and different microbes possible (iv) Possible to detect individual cells when ribosomal RNA is target (v) It can combine with automated scanning machines that filter surfaces for fluorescent objects	(i) Monitoring is purely taxonomic and requires expensive facilities (ii) Difficult to create a particular and unequivocally restricted probe for a certain class of microbes (iii) A sluggish and complicated procedure due to involvement of an elaborate hybridization procedure for a specific probe
Polymerase chain reaction -based techniques [13]	(i) Rapid, flexible, and cost-effective (ii) Sensitive and selective (iii) Detection of VBNC state (iv) Indirect detection of many pathogens possible (v) RT-PCR technique allows assessing the viability of the cells	(i) Long reaction time (ii) Need adequate amounts of nucleotides from the targeted bacterium (iii) Some prior information is required to design primer (iv) Technical expert is required as it is prone to error and contamination (v) Limited information on a pathogen’s infectiousness
Loop-mediated Isothermal Amplification [62]	(i) Stable, simple and specific (ii) Non-target DNA do not affect the DNA amplification (iii) Amplification takes place at isothermal conditions, so simple heating device is enough (iv) Applicable to RNA also by employing reverse transcriptase	(i) Carry-over contamination is possible, leading to false-positive results (ii) Complex primer design (iii) Multiplex amplification is challenging
DNA Microarray [63]	(i) Improve the selectivity significantly (ii) High throughput analysis possible (iii) Rapid-results within 2–4 h (iv) Relatively low cost	(i) Absolute quantification is difficult (ii) Difficult to confirm viability of microorganisms (iii) Require highly skilled personnel, specialized and expensive infrastructure
Next Generation Sequencing [64]	(i) Capable of massive parallel sequencing (ii) Quantitative and sensitive detection of genomic aberrations (iii) Applicable to a wide range of molecular biology	(i) Need to re-confirm the results with Sanger sequencing methods for clinical applications (ii) Homopolymer bias/errors (iii) High complexity of workflow and results
Enzymatic Method		
Enzymatic Method [65]	(i) Simple, fast (1 h), no trained staff or advanced tools, required (ii) Highly selective and sensitive (iii) Screening tests could be conducted without even any cultivation steps	(i) Enzymes are generally expensive and lose activity easily (ii) Any fluorescence signal enhancement techniques require prior growth of the target microbes (iii) Compatibility issue
